# 

**Published:** 1875-06-15

**Authors:** 


*ORIGINAL COMMUNICATIONS:*

The Verdict of Science concerning the Effects of Alcohol on Man. By N. S. Davis, M. D. 327

*TRANSLATIONS:*

Clinical Thermometry. By Dr. B. Kraus. Condensed and Translated by Dr. H. Gradle, 333

*EDITORIAL DEPARTMENT:*

The Study of Ætiology; Æsculapian Society of the Wabash Valley 337

Prize Essay—Medical Association of the State of Alabama; The Boston Medical and Surgical Journal; American Medical Association 338

*CORRESPONDENCE:*

A Letter from Paris 339

*GLEANINGS FROM OUR EXCHANGES:*

A Case of Unilateral Sweating of the Head. By Alonzo Bryan, M. D. 344

A Novel Treatment of Obstinate Vomiting in Pregnancy. By Edward Copeman, M. D., F. R. C. P. 345

*BOOK REVIEWS* 346

